# Equivalence of Informations Characterizes Bregman Divergences

**DOI:** 10.3390/e27070766

**Published:** 2025-07-19

**Authors:** Philip S. Chodrow

**Affiliations:** Department of Computer Science, Middlebury College, Middlebury, VT 05753, USA; pchodrow@middlebury.edu

**Keywords:** Bregman divergence, mutual information, characterization theorem, convexity

## Abstract

Bregman divergences form a class of distance-like comparison functions which plays fundamental roles in optimization, statistics, and information theory. One important property of Bregman divergences is that they generate agreement between two useful formulations of information content (in the sense of variability or non-uniformity) in weighted collections of vectors. The first of these is the Jensen gap information, which measures the difference between the mean value of a strictly convex function evaluated on a weighted set of vectors and the value of that function evaluated at the centroid of that collection. The second of these is the divergence information, which measures the mean divergence of the vectors in the collection from their centroid. In this brief note, we prove that the agreement between Jensen gap and divergence informations in fact characterizes the class of Bregman divergences; they are the only divergences that generate this agreement for arbitrary weighted sets of data vectors.

## 1. Introduction

For a convex set $C\subseteq R^{\ell }$ with relative interior $C_{*}$ and a strictly convex function ϕ:C→R differentiable on $C_{*}$, the Bregman divergence induced by ϕ is the function $d_{\phi }:C\times C_{*}\to R$ defined by

$$\begin{array}{c} d_{\phi }\left(x_{1},x_{2}\right)=\phi \left(x_{1}\right)-\phi \left(x_{2}\right)-\nabla \phi {\left(x_{2}\right)}^{T}\left(x_{1}-x_{2}\right). \end{array}$$

Two common examples of Bregman divergences are:

- The squared Mahalanobis distance $d_{\phi }\left(x_{1},x_{2}\right)={\left(x_{1}-x_{2}\right)}^{T}W\left(x_{1}-x_{2}\right)$, where W is a positive-definite matrix. The function ϕ is given by $\phi \left(x\right)=\frac{1}{2}x^{T}Wx$. The special case W=I gives the squared Euclidean distance. This divergence may be defined on $C=R^{\ell }$.
- The Kullback–Leibler (KL) divergence $d_{\phi }\left(x,y\right)=\sum_{j=1}^{\ell }x_{j}\log\frac{x_{j}}{y_{j}}$, where x and y are probability vectors. Here, C is the simplex $\Delta_{\ell }=\left\{x\in R^{\ell }\;|\;\sum_{j=1}^{\ell }x_{j}=1,\;x_{j}\geq 0\;\forall j\right\}$. The KL divergence is induced by the negative entropy function $\phi \left(x\right)=\sum_{j=1}^{\ell }x_{j}\log x_{j}$. This divergence can be extended to general convex subsets of $R_{+}^{\ell }$ with formula $d_{\phi }\left(x,y\right)=\sum_{j=1}^{\ell }\left(x_{j}\log\frac{x_{j}}{y_{j}}+y_{j}-x_{j}\right)$ for $x,y\in R_{+}^{\ell }$. When computing the KL divergence, we use the convention 0log0=0.

While it is possible to extend the definition of Bregman divergences to Banach spaces [1], in this note we focus on divergences whose domains are convex subsets of $R^{\ell }$. In this setting, it is possible to interpret the Bregman divergence as a comparison between the difference $\phi \left(x_{1}\right)-\phi \left(x_{2}\right)$ on the one hand and the linearized approximation of this difference about $x_{2}$ given by $\nabla \phi {\left(x_{2}\right)}^{T}\left(x_{1}-x_{2}\right)$ on the other.

Like metrics, Bregman divergences are positive-definite: $d_{\phi }\left(x,y\right)\geq 0$ with equality if and only if x=y. Unlike metrics, Bregman divergences are not in general symmetric and do not in general satisfy a triangle inequality, though they do satisfy a “law of cosines” and a generalized Pythagorean theorem [2]. Bregman divergences are locally distance-like in that they induce a Riemannian metric on C obtained by the small-δ expansion

$$\begin{array}{c} d_{\phi }\left(x+\delta ,x\right)=\frac{1}{2}\delta^{T}H\phi \left(x\right)\delta +o\left({∥\delta ∥}^{2}\right), \end{array}$$

where δ is a small perturbation vector and Hϕ(x) is the Hessian of ϕ at x. Because ϕ is strictly convex, Hϕ(x) is positive-definite and defines a Riemannian metric on C [3]; much work in information geometry [4] pursues the geometry induced by this metric and its connections to statistical inference. Bregman divergences [5] also play fundamental roles in machine learning, optimization, and information theory. They are the unique class of distance-like losses for which iterative, centroid-based clustering algorithms (such as *k*-means) always reduce the global loss [2,6] Bregman divergences are central in the formulation of mirror-descent methods for convex optimization [7] and have a connection via convex duality to Fenchel-Young loss functions [4,8]. See Reem et al. [9] for a more detailed review of Bregman divergences.

Bregman divergences provide one natural route through which to generalize Shannon information theory, with the differentiable function −ϕ taking on the role of the Shannon entropy. Indeed, generalized entropies play a role in describing the the asymptotic performance of learning algorithms; there exist a number of inequalities relating Bregman divergences to these generalized entropies [10,11]. Multiple characterization theorems exist for many information-theoretic quantities, including entropy [12,13,14], mutual information [15,16], and the Kullback–Leibler divergence [17,18]. This author, however, is aware of only one extant characterization of the class of Bregman divergences, due to Banerjee et al. [6]: Bregman divergences are the unique class of loss functions that render conditional expectations uniquely loss-minimizing in stochastic prediction problems. This characterization is the foundation of the connection between Bregman divergences and iterative centroid-based clustering algorithms noted above.

In this short note, we prove a new characterization of the class of Bregman divergences. This characterization is based on an equality of two common formulations of information content in weighted collections of finite-dimensional vectors.

## 2. Bregman Divergences Relate Two Informations

Let $\mu \in \Delta_{n}$ be a probability measure over *n* points $x_{1},\ldots ,x_{n}\in C$. We collect these points into a matrix X, and in a small abuse of notation, we consider this matrix to be an element of $C^{n}$. We now define two standard formulations of information, each of which we consider as a function $\Delta_{n}\times C^{n}\to R$. The first formulation compares a weighted sum of strictly convex loss function evaluations on data points to the same loss function evaluated at the data centroid.

**Definition 1**  (Jensen Gap Information)**.** *Let ϕ:C→R be a strictly convex function on C. The *Jensen gap information* is the function $I_{\phi }:\Delta_{n}\times C^{n}\to R$ given by*

$$\begin{array}{c} I_{\phi }\left(\mu ,X\right)≜\sum_{i=1}^{n}\mu_{i}\phi \left(x_{i}\right)-\phi \left(y\right), \end{array}$$

*where $y=\sum_{i=1}^{n}\mu_{i}x_{i}$.*

If we define *X* to be a random vector that takes value $x_{i}$ with probability $\mu_{i}$, Jensen’s inequality states that E[ϕ(X)]≥ϕ(E[X]), with equality holding only if *X* is constant (i.e., if there exists *i* such that $\mu_{i}=1$). The Jensen gap information is a measure of the difference of the two sides of this inequality; indeed, $E\left[\phi \left(X\right)\right]=\phi \left(E\left[X\right]\right)+I_{\phi }\left(\mu ,X\right)$ [2,6]. This formulation makes clear that $I_{\phi }$ is non-negative and that $I_{\phi }\left(\mu ,X\right)=0$ if and only if $x_{i}=x_{j}$ whenever $\mu_{i}>0$ and $\mu_{j}>0$.

Another standard formulation expresses information content as a weighted mean of divergences of data points from their centroid.

**Definition 2**  (Divergence)**.** *A function d:C×C→R is a *divergence* if $d(x_{1},x_{2})\geq 0$ for any $x_{1},x_{2}\in C$, with equality if and only if $x_{1}=x_{2}$.*

**Definition 3**  (Divergence Information)**.** *Let d be a divergence. The *divergence information* is the function $I_{d}:\Delta_{n}\times C^{n}\to R$ given by*

(1) $$\begin{array}{c} I_{d}\left(\mu ,X\right)≜\sum_{i=1}^{n}\mu_{i}d\left(x_{i},y\right)\;, \end{array}$$

*where $y=\sum_{i=1}^{n}\mu_{i}x_{i}$.*

In this definition, we assume that $y\in C_{*}$; as noted by Banerjee et al. [2], this assumption is not restrictive since the set C can be replaced with the convex hull of the data X without loss of generality. The divergence information measures the μ-weighted average divergence of $x_{i}$ from the centroid y. This divergence information is related to the characterization result for Bregman divergences by Banerjee et al. [6]: a divergence *d* is a Bregman divergence if and only if the vector $y=\sum_{i=1}^{n}\mu_{i}x_{i}$ is the unique minimizer of the function $\sum_{i=1}^{n}\mu_{i}d\left(x_{i},\cdot \right)$ appearing on the righthand side of Equation (1) for any choice of μ and X.

There are several important cases in which the Jensen gap information and the divergence information coincide.

**Definition 4**  (Information Equivalence)**.** *We say that a pair (ϕ,d) comprising a strictly convex function ϕ:C→R and a divergence d:C×C→R satisfies the *information equivalence property* if, for all $\left(\mu ,X\right)\in \Delta_{n}\times C^{n}$, it holds that*

(2) $$\begin{array}{c} I_{\phi }\left(\mu ,X\right)=I_{d}\left(\mu ,X\right)\;. \end{array}$$

**Figure 1:**
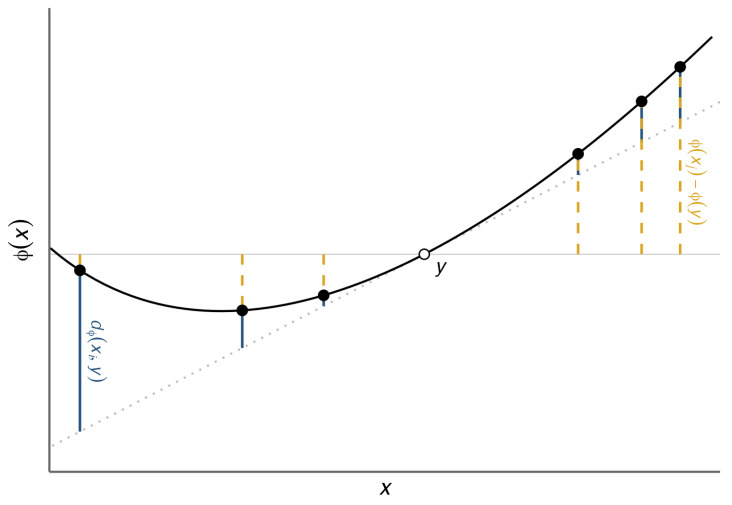
Illustration of the information equivalence property in one dimension. Several data points $x_{i}$ are shown (black) alongside their centroid $y=\sum_{i=1}^{n}\mu_{i}x_{i}$ (hollow). The dashed grey line gives the tangent to ϕ at *y*, with equation $t\left(x\right)=\phi \left(y\right)+\phi^{\prime }\left(y\right)\left(x-y\right)$. Dashed yellow segments give the value of $\phi \left(x_{i}\right)-\phi \left(y\right)$; the *signed* weighted mean of the lengths of these segments is the Jensen-gap information $I_{\phi }\left(\mu ,X\right)$. Solid blue segments give the value of $d_{\phi }\left(x_{i},y\right)$; the *unsigned* weighted mean of the lengths of these segments is the divergence information $I_{d}\left(\mu ,X\right)$. Information equivalence (2) asserts these two weighted means are equal. For the purposes of this visualization, μ is the uniform distribution $\mu_{i}=\frac{1}{n}$. The function ϕ shown is ϕ(x)=xlogx.

A graphical illustration of information equivalence is shown in Figure 1.

**Lemma 1**  (Information Equivalence with Bregman Divergences [2,6])**.** *The pair $\left(\phi ,d_{\phi }\right)$ satisfies the information equivalence property.*

The proof is a direct calculation and is provided by Banerjee et al. [2]. When $\phi \left(x\right)=\frac{1}{2}{∥x∥}^{2}$ and $d=d_{\phi }$ is the squared Euclidean distance, the information equivalence property (2) is the identity

(3) $$\begin{array}{c} \sum_{i=1}^{n}\mu_{i}{∥x_{i}∥}^{2}-{∥\sum_{i=1}^{n}\mu_{i}x_{i}∥}^{2}=\sum_{i=1}^{n}\mu_{i}{∥x_{i}-\sum_{i=1}^{n}\mu_{i}x_{i}∥}^{2}\;. \end{array}$$

The righthand side of (3) is the weighted sum-of-squares loss of the data points $x_{i}$ with respect to their centroid $\sum_{i=1}^{n}\mu_{i}x_{i}$, which is often used in statistical tests and clustering algorithms. Equation (3) asserts that this loss may also be computed from a weighted average of the norms of the data points.

When C is the probability simplex, $\phi \left(x\right)=\sum_{i=1}^{n}x_{i}\log x_{i}$ is the negative entropy, and $d=d_{\phi }$ is the KL divergence, the information equivalence property (2) expresses the equality of two equivalent formulations of the mutual information for discrete random variables. Let *A* and *B* be discrete random variables on alphabets A of size *n* and B of size *ℓ*, respectively. Suppose that their joint distribution is $p_{A,B}\left(a_{i},b_{j}\right)=\mu_{i}x_{ij}$. Let y be the vector with entries $y_{j}=\sum_{i=1}^{n}\mu_{i}x_{ij}$; then, y is the marginal distribution of *B*. The Jensen gap information $I_{\phi }\left(\mu ,X\right)$ is

$$\begin{array}{cc} I_{\phi }\left(\mu ,X\right) & =\underset{-H(B|A)}{\underset{︸}{\sum_{i=1}^{n}\mu_{i}\sum_{j=1}^{\ell }x_{ij}\log x_{ij}}}-\underset{-H(B)}{\underset{︸}{\sum_{j=1}^{\ell }y_{j}\log y_{j}}}; \end{array}$$

which expresses the mutual information I(A;B) between random variables *A* and *B* in the entropy-reduction formulation, I(A;B)=H(B)−H(B|A) [19]. On the other hand, the divergence information $I_{d}\left(\mu ,X\right)$ is

$$\begin{array}{c} I_{d}\left(\mu ,X\right)=\sum_{i=1}^{n}\mu_{i}\underset{d_{\phi }\left(x_{i},y\right)}{\underset{︸}{\sum_{j=1}^{\ell }x_{ij}\log\frac{x_{ij}}{y_{j}},}} \end{array}$$

which expresses the mutual information I(A;B) instead as the weighted sum of KL divergences of $x_{i}$ from y.

Our contribution in this paper is to prove a converse to Lemma 1: the Bregman divergence $d_{\phi }$ is the *only* divergence that satisfies information equivalence with ϕ.

## 3. Main Result

**Theorem 1.**  If the pair (ϕ,d) satisfies the information equivalence property *(2)*, then d is the Bregman divergence induced by ϕ: $d\left(x,y\right)=d_{\phi }\left(x,y\right)$ for any x∈C and $y\in C_{*}$.

Let (ϕ,d) satisfy information equivalence (2). For any x∈C and $y\in C_{*}$, we can write

(4) $$\begin{array}{c} d(x,y)=\phi (x)-\phi (y)+f(x,y) \end{array}$$

for some unknown function $f:C\times C_{*}\to R$. We aim to show that $f\left(x,y\right)=-\nabla \phi {\left(y\right)}^{T}\left(x-y\right)$ for all x∈C and $y\in C_{*}$.

Our first step is to show that *f* is an affine function of its first argument x on C. To do so, we observe that if $\mu \in \Delta_{n}$ and $X\in C^{n}$ are such that $\sum_{i=1}^{n}\mu_{i}x_{i}=y$, then we have

$$\begin{array}{cc} \sum_{i=1}^{n}\mu_{i}\phi \left(x_{i}\right)-\phi \left(y\right) & =\sum_{i=1}^{n}\mu_{i}d\left(x_{i},y\right)\\ \; & =\sum_{i=1}^{n}\mu_{i}\left[\phi \left(x_{i}\right)-\phi \left(y\right)+f\left(x_{i},y\right)\right]\\ \; & =\sum_{i=1}^{n}\mu_{i}\phi \left(x_{i}\right)-\phi \left(y\right)+\sum_{i=1}^{n}\mu_{i}f\left(x_{i},y\right), \end{array}$$

where the first line follows from information equivalence. It follows that

(5) $$\begin{array}{c} \sum_{i=1}^{n}\mu_{i}f\left(x_{i},y\right)=0. \end{array}$$

Fix $y\in C_{*}$. Let $A_{y}=\left\{v\in R^{n}\;|\;y+v\in C\right\}$, and for any ϵ>0 let $B_{y}\left(\epsilon \right)=A_{y}\cap \left\{v\in R^{n}\;|\;∥v∥<\epsilon \right\}$. Pick ϵ>0 sufficiently small that, for all $v\in B_{y}\left(\epsilon \right)$, it holds that both y+v∈C and y−v∈C; this is possible due to the relative openness of $C_{*}$. For notational compactness, let $B_{y}=B_{y}\left(\epsilon \right)$. Since $B_{y}$ is the intersection of a Euclidean ball with convex set $A_{y}$, it is also convex.

Consider the function $g_{y}:A_{y}\to R$ given by $g_{y}\left(v\right)=f\left(v+y,y\right)$. The condition (5) implies that

(6) $$\begin{array}{c} \sum_{i=1}^{n}\mu_{i}g_{y}\left(v_{i}\right)=0\;. \end{array}$$

for any $v_{1},\ldots ,v_{n}\in A_{y}$ such that $\sum_{i=1}^{n}\mu_{i}v_{i}=0$.

To show that f(·,y) is affine, it suffices to show that the function $g_{y}$ is linear on $A_{y}$. We do this through two short lemmas. In each, we characterize the behavior of $g_{y}$ on the relative ball $B_{y}$ before extending this characterization to the entire domain $A_{y}$.

**Lemma 2.**  *For any vector $v\in A_{y}$ and scalar α such that $\alpha v\in A_{y}$, we have $g_{y}\left(\alpha v\right)=\alpha g_{y}\left(v\right)$.*

**Proof.**  We will first prove the lemma in the restricted case that α=−1 and $v\in B_{y}$. By Equation (6), we have that

$$\begin{array}{c} \frac{1}{2}g_{y}\left(v\right)+\frac{1}{2}g_{y}\left(-v\right)=0\;. \end{array}$$

from which it follows that $g_{y}\left(-v\right)=-g_{y}\left(v\right)$. Let us now assume that $v\in B_{y}$ but that α is general; we will then use this to prove the more general setting $v\in A_{y}$. We proceed by cases.

1. α=0. The previous argument implies that $g_{y}\left(0\right)=0$.
2. α>0. Since $\frac{\alpha }{1+\alpha }\left(-v\right)+\frac{1}{1+\alpha }\left(\alpha v\right)=0$, an application of Equation (6) gives $\alpha g_{y}\left(-v\right)+g_{y}\left(\alpha v\right)=0$; isolating $g_{y}\left(\alpha v\right)$ and applying the previous argument proves the case.
3. α<0. This case follows by applying the proof of the previous case, replacing α with −α.

Now, assume only that $v\in A_{y}$. Choose β>0 so that $\beta v\in B_{y}$ and $\beta \alpha v\in B_{y}$; $\beta =\min\left\{\frac{\epsilon }{2\alpha ∥v∥},1\right\}$ is one sufficient choice. Then, by our previous argument, we have $g_{y}\left(v\right)=g_{y}\left(\frac{1}{\beta }\beta v\right)=\frac{1}{\beta }g_{y}\left(\beta v\right)$, from which we infer $g_{y}\left(\beta v\right)=\beta g_{y}\left(v\right)$. Using this, we can compute $g_{y}\left(\alpha v\right)=g_{y}\left(\frac{1}{\beta }\beta \alpha v\right)=\frac{\alpha }{\beta }g_{y}\left(\beta v\right)=cg_{y}\left(v\right)$, which proves the lemma. □

**Lemma 3.**  *The function $g_{y}$ is linear on $A_{y}$: for any $\alpha \in R^{n}$ and vectors $v_{1},\ldots ,v_{n}\in A_{y}$ such that $\sum_{i=1}^{n}\alpha_{i}v_{i}\in A_{y}$, it holds that*

$$\begin{array}{c} g_{y}\left(\sum_{i=1}^{n}\alpha_{i}v_{i}\right)=\sum_{i=1}^{n}\alpha_{i}g_{y}\left(v_{i}\right)\;. \end{array}$$

**Proof.**  Let us first assume that $\alpha \in \Delta_{n}$ and $v_{1},\ldots ,v_{n}\in B_{y}$. Applying Equation (6) gives

$$\begin{array}{c} \frac{1}{2}g_{y}\left(\sum_{i=1}^{n}\alpha_{i}v_{i}\right)+\frac{1}{2}\sum_{i=1}^{n}\alpha_{i}g_{y}\left(-v_{i}\right)=0\;, \end{array}$$

from which applying Lemma 2 gives the result under these hypotheses.

We now consider the general case. For each *i*, choose $\beta_{i}\neq 0$ so that $\beta_{i}\alpha_{i}>0$ and ${\tilde{v}}_{i}≜\beta_{i}v_{i}\in B_{y}$. Let $M=\sum_{i=1}^{n}\frac{\alpha_{i}}{\beta_{i}}$. Define the vector $\tilde{\alpha }\in \Delta_{n}$ with entries ${\tilde{\alpha }}_{i}=\frac{\alpha_{i}}{M\beta_{i}}$. Then, by construction, $\alpha_{i}v_{i}=M{\tilde{\alpha }}_{i}{\tilde{v}}_{i}$ for each *i*. Applying Lemma 2 and the restricted case above, we can then compute

$$\begin{array}{cc} g_{y}\left(\sum_{i=1}^{n}\alpha_{i}v_{i}\right) & =g_{y}\left(\sum_{i=1}^{n}M{\tilde{\alpha }}_{i}{\tilde{v}}_{i}\right)\\ \; & =Mg_{y}\left(\sum_{i=1}^{n}{\tilde{\alpha }}_{i}{\tilde{v}}_{i}\right)\\ \; & =M\sum_{i=1}^{n}{\tilde{\alpha }}_{i}g_{y}\left({\tilde{v}}_{i}\right)\\ \; & =M\sum_{i=1}^{n}{\tilde{\alpha }}_{i}\beta_{i}g_{y}\left(v_{i}\right)\\ \; & =\sum_{i=1}^{n}\alpha_{i}g_{y}\left(v_{i}\right);, \end{array}$$

which completes the proof. □

**Proof of Theorem 1.**  Fix $y\in C_{*}$. The preceding lemmas prove that the function $g_{y}$ is linear on $A_{y}$. Since for constant y the function *f* in (4) is a translation of $g_{y}$ in its first argument, it follows that *f* is affine as a function of its first argument x. We may therefore write, for all x∈C and $y\in C_{*}$,

(7) $$\begin{array}{c} f\left(x,y\right)=h_{1}{\left(y\right)}^{T}x+h_{2}\left(y\right)\;. \end{array}$$

for some functions $h_{1}:C_{*}\to R^{\ell }$ and $h_{2}:C_{*}\to R$.

We now determine these functions. First, since ϕ is differentiable on $C_{*}$ and f(x,y) is affine in x, d(x,y) is differentiable in its first argument on $C_{*}$. Since *d* is a divergence, y is a critical point of the function d(·,y) on $C_{*}$. It follows that $\nabla_{1}d\left(y,y\right)$, the gradient of *d* with respect to its first argument, is orthogonal to $C_{*}$ at y:

(8) $$\begin{array}{c} \nabla_{1}d{\left(y,y\right)}^{T}\left(x-y\right)=0 \end{array}$$

for any x∈C. We can compute $\nabla_{1}d\left(y,y\right)$ explicitly; it is $\nabla_{1}d\left(y,y\right)=\nabla \phi \left(y\right)+h_{1}\left(y\right)$, which combined with (8) gives

(9) $$\begin{array}{c} {\left(\nabla \phi \left(y\right)+h_{1}\left(y\right)\right)}^{T}\left(x-y\right)=0\;. \end{array}$$

for any x∈C and $y\in C^{*}$.

Now, the condition that d(y,y)=0 implies that $h_{2}\left(y\right)=-h_{1}{\left(y\right)}^{T}y$. Using Equations (9) and (7), we then compute

$$\begin{array}{cc} -\nabla \phi {\left(y\right)}^{T}\left(x-y\right) & =h_{1}{\left(y\right)}^{T}\left(x-y\right)\\ \; & =h_{1}{\left(y\right)}^{T}x+h_{2}\left(y\right)\\ \; & =f(x,y). \end{array}$$

Recalling the definition of *f* in (4), we conclude that

$$\begin{array}{c} d\left(x,y\right)=\phi \left(x\right)-\phi \left(y\right)-\nabla \phi {\left(y\right)}^{T}\left(x-y\right), \end{array}$$

which is the Bregman divergence induced by ϕ. This completes the proof. □

## 4. Discussion

We have shown that the class of Bregman divergences is the unique class of divergences that induce agreement between the Jensen gap and divergence informations. This result offers some further perspective on the role for Bregman divergences in data clustering and quantization [2]. The Jensen gap information $I_{\phi }$ is a natural loss function for such tasks, with one motivation as follows. Suppose that we wish to measure the complexity of a set of data points X with weights $\mu \in \Delta_{n}$ using a weighted per-observation loss function and a term that depends only on the centroid $y=\sum_{i=1}^{n}\mu_{i}x_{i}$ of the data:

$$\begin{array}{c} L\left(\mu ,X\right)=\sum_{i=1}^{n}\mu_{i}\psi \left(x_{i}\right)+\rho \left(y\right). \end{array}$$

A natural stipulation for the loss function *L* is that replacing two data points $x_{1}$ and $x_{2}$ with their weighted mean $x=\frac{\mu_{1}}{\mu_{1}+\mu_{2}}x_{1}+\frac{\mu_{2}}{\mu_{1}+\mu_{2}}x_{2}$ should strictly decrease the loss when $x_{1}\neq x_{2}$; this requirement is equivalent to strict convexity of the function ψ. If we further require that L(μ,X)=0 when each row of X is identical, we find that ρ(y)=−ψ(y) and that our loss function is the Jensen gap information: $L\left(\mu ,X\right)=I_{\psi }\left(\mu ,X\right)$. The present result shows that this natural formulation fully determines the choice of how to perform pairwise comparisons between individual data points; only the corresponding Bregman divergence can serve as a positive-definite comparator that is consistent with the Jensen gap information.

An extension of this result to the setting of Bregman divergences defined on more general spaces, such as Banach spaces [1], would be of considerable interest for problems in functional data clustering [20].

## Data Availability

No new data were created or analyzed in this study. Data sharing is not applicable to this article.

## References

[1] Bauschke H.H., Borwein J.M., Combettes P.L. (2003). Bregman Monotone Optimization Algorithms. SIAM J. Control Optim..

[2] Banerjee A., Merugu S., Dhillon I.S., Ghosh J. (2005). Clustering with Bregman divergences. J. Mach. Learn. Res..

[3] Amari S., Cichocki A. (2010). Information Geometry of Divergence Functions. Bull. Pol. Acad. Sci. Tech. Sci..

[4] Amari S. (2016). Information Geometry and Its Applications.

[5] Bregman L. (1967). The Relaxation Method of Finding the Common Point of Convex Sets and Its Application to the Solution of Problems in Convex Programming. USSR Comput. Math. Math. Phys..

[6] Banerjee A., Guo X., Wang H. (2005). On the Optimality of Conditional Expectation as a Bregman Predictor. IEEE Trans. Inf. Theory.

[7] Nemirovsky A., Yudin D. (1983). Problem Complexity and Method Efficiency in Optimization.

[8] Blondel M., Martins A.F.T., Niculae V. (2020). Learning with Fenchel-Young Losses. J. Mach. Learn. Res..

[9] Reem D., Reich S., De Pierro A. (2019). Re-Examination of Bregman Functions and New Properties of Their Divergences. Optimization.

[10] Painsky A., Wornell G.W. (2020). Bregman Divergence Bounds and Universality Properties of the Logarithmic Loss. IEEE Trans. Inf. Theory.

[11] Xu A. (2021). Continuity of Generalized Entropy and Statistical Learning. arXiv.

[12] Baez J.C., Fritz T., Leinster T. (2011). A Characterization of Entropy in Terms of Information Loss. Entropy.

[13] Faddeev D.K. (1956). On the Concept of Entropy of a Finite Probabilistic Scheme. Uspekhi Mat. Nauk.

[14] Shannon C.E. (1948). A Mathematical Theory of Communication. Bell Syst. Tech. J..

[15] Fullwood J. (2023). An Axiomatic Characterization of Mutual Information. Entropy.

[16] Frankel D.M., Volij O. (2011). Measuring School Segregation. J. Econ. Theory.

[17] Jiao J., Courtade T., No A., Venkat K., Weissman T. (2014). Information Measures: The Curious Case of the Binary Alphabet. IEEE Trans. Inf. Theory.

[18] Hobson A. (1969). A New Theorem of Information Theory. J. Stat. Phys..

[19] Cover T.M., Thomas J.A. (2012). Elements of Information Theory.

[20] Zhang M., Parnell A. (2023). Review of Clustering Methods for Functional Data. ACM Trans. Knowl. Discov. Data.

